# A Descriptive Evaluation of Evidence‐Based Rounds in Critical Care Using Mixed Data Types

**DOI:** 10.1111/jan.70420

**Published:** 2025-12-12

**Authors:** P. Yerbury, S. Sutherland, T. Venes, A. Nair, J. Ede

**Affiliations:** ^1^ Oxford University Hospital NHS Foundation Trust Oxford UK; ^2^ Oxford Brookes University Oxford UK; ^3^ University of Exeter Exeter UK

**Keywords:** critical care, education, health services research, leadership, research implementation, research in practice

## Abstract

**Objectives:**

To pilot and evaluate the implementation of a structured Evidence‐Based Rounds (EBR) education model in critical care.

**Design:**

A mixed data type design was used to evaluate Evidence‐Based Rounds in a critical care setting. Structured observational data were captured and open‐ended survey responses were submitted by attendees. Content analysis and descriptive statistics were used to analyse survey findings.

**Results:**

Seventeen rounds were completed between March 2023 and January 2024 with a total of *n* = 83 clinical staff members. From these, *n* = 55 staff completed and submitted evaluation surveys. Rounds were most frequently attended by nurses of all bandings including senior clinical nurses, support workers and student nurses. Evidence‐Based Rounds were globally perceived as a positive and useful education strategy and staff were very willing to attend future sessions. Patient outcomes were not directly assessed and rounds specifically facilitated three outcomes: (1) helping staff apply evidence to practice, (2) building staff confidence in presenting clinical information and (3) supporting staff in identifying local improvements to patient care.

**Conclusion:**

Evidence‐Based Rounds are an adaptable effective model of bedside education within critical care. In our setting, staff perceived that this model facilitated the application of evidence in clinical practice and positively influenced feelings of confidence. Importantly, this education strategy empowered nurses to explore and identify improvements locally to patient care. Whilst this model offers a practical education approach to address some of the key critical care workforce issues, such as an expanding curriculum and loss of senior staff, it could also be widely adopted to other clinical areas.

**Implications for the Profession:**

Evidence‐Based Rounds are perceived by staff as a successful bedside education model that facilitates nurses to apply evidence in practice. It is feasible that this strategy is a potentially sustainable, low‐cost model for continuing professional development centred around routine clinical work.

**Patient and Public Contribution:**

No patient or public contribution.

## Summary

- There is an expanding critical care education curriculum and a reduction in senior nurses to support workplace learning for staff.
- Evidence‐Based Rounds are perceived as an effective and adaptable bedside education model, which critical care nurses found to be highly effective, engaging and contributed to their confidence in applying evidence‐based care.
- The Evidence‐Based Rounds model could be applied to other critical care settings and ward areas, which this manuscript could support in terms of structure, delivery and evaluation.

## 1 Introduction

Evidence‐based medicine (EBM) originated in the literature more than 20 years ago (Dusin et al. 2023). However, its beginnings can be traced much earlier to Hippocrates who advocated for observation and systematic study in medicine. A recent and more inclusive framework is evidence‐based practice (EBP), which encompasses other health professionals using research and evidence to underpin care delivery and evaluation (Aitken et al. 2011; Schaffer et al. 2013; Dusin et al. 2023). This clinical framework is characterised by a lifelong problem‐solving approach that integrates the best available evidence with clinical expertise to address patient‐centred needs and improve patient outcomes (Melnyk et al. 2011).

Implementing EBP in all clinical settings is critical to patient experience and outcomes yet challenging to achieve effectively (Dusin et al. 2023). To address this, Sackett et al. proposed a pragmatic model operationalising EBP and suggested five core elements: (1) asking a question, (2) acquiring the best evidence, (3) appraising the evidence, (4) applying the findings and (5) evaluating outcomes of change (Sackett 1997). Since this, there have been approximately 18 other frameworks developed across heterogenic clinical settings and populations with varying success (Dusin et al. 2023). A recent scoping review highlights the Iowa EBP model as particularly effective due to its emphasis on interprofessional collaboration, widespread validation and availability of supporting documentation (Dusin et al. 2023). A central tenet of this model is identifying change needs in the clinical environment and having the ability to critically appraise literature (Buckwalter et al. 2017).

In the United Kingdom (UK), Intensive Care Units (ICU) prioritise EBP to deliver evidence‐driven interventions and treatments crucial for the sustainability and efficiency of the National Health Service (NHS) (Intensive Care Society 2016; ICS 2022). Educating critical care staff in EBP, through formal education programmes, mentorship and continuous engagement with research is a high priority (McGivern et al. 2025). However, the current ICU workforce faces unique challenges such as a rapidly expanding curriculum, high patient acuity and a shortage of senior nursing staff (Critical Care Network Lead Nurses group 2022). A recent survey of members of the UK CC3N found that one in two current adult critical care nurses are expecting to leave their current role in the next 3 years (Critical Care Network Lead Nurses group 2022). This anticipated turnover will lead to a workforce with greater educational needs and fewer experienced staff to provide bedside mentorship and training (McGivern et al. 2025). Ultimately, this could compromise the safe delivery of high‐quality patient care (ICS 2022). Given the limited opportunities for formalised workplace learning within the current national workforce landscape, critical care environments need to adapt traditional staff education by incorporating innovative and diverse, but complementary bedside approaches (Tobiano et al. 2019).

Workplace learning is preferred by nurses and continuing professional development (CPD) is most effective when it is work related (Jones et al. 2009). Rounds, such as the traditional ward round where patients are reviewed by multiple clinical experts, provide additional opportunities to frame bedside teaching and learning within a clinical area whilst delivering expertise (Dalmaso et al. 2015). Other more inclusive models such as interdisciplinary bedside rounds (IBR) or Evidence in Action (EIA) rounds can be used to bridge the gap between theory and evidence‐based practice to improve clinical care. The use of ward rounds or grand rounds is also well‐established in health care (Schulte et al. 2012; Heip et al. 2020; El Atty et al. 2022).

An adaptation to traditional nursing rounds, known as Evidence‐Based Rounds (EBR), aims to formalise social and real‐time learning by creating an inquisitive dialogue between multiple staff to explore care and evidence for carefully selected patient case studies, which can then be applied directly to patient care (Tobiano et al. 2019). Drawing on workplace learning theory, EBR allows staff to participate in learning whilst applying evidence directly to patient care. This conceptual lens provides a rationale for EBR's potential suitability in clinical settings, where bedside learning can facilitate knowledge translation (Teunissen 2015; Verhees et al. 2023). These various rounding models shift learning towards a discussion‐based approach in contrast to a traditional classroom‐based setting and promote ‘active’ learning, which enhances continuous development (Lees and Meyer 2011). Importantly, after a round of purposeful review that includes the patient and integrates research, the quality of care given to patients improves (Schulte et al. 2012; Heip et al. 2020).

Existing ICU education models do not fully address workforce issues within the current context, and there is a need to explore adaptable, scalable and resource‐efficient educational strategies such as EBR. There is limited evidence on how structured EBR models can be sustained over time and integrated into routine clinical practice. Understanding the perceived benefits is crucial for scaling up this educational approach and increasing organisational support. The aim of this study was to conduct an exploratory, single‐site pilot and evaluate the implementation of a structured EBR education model in a critical care setting.

## 2 Methods

### 2.1 Study Design

A multiple methods design was used to evaluate the EBR education model utilising direct observational data and survey evaluation responses, between March 2023 and January 2024. This manuscript was reported as per the SQUIRE checklist (Ogrinc et al. 2008) (Data S1). Observational and survey data were considered appropriate methods to capture the impact of EBR. Observations allowed the researchers to collect descriptive round data as well as capture interactions between staff and the facilitator, focusing more on the social‐educational interactions between staff. Survey data ensured that the perceptions of attendees were present within the EBR evaluation data. We did not collect patient‐related outcomes resulting from this work, as the primary aim of this pilot was to examine the feasibility of this education model within an ICU setting.

### 2.2 Evidence‐Based Round Model

#### 2.2.1 Structure

An EBR is a group learning exercise that focusses on identifying the evidence base for a particular patient case study in real time. Rounds were undertaken no more than twice a week and lasted for no longer than an hour each. Critical care staff were notified a few hours before that a round would take place that day (between 3 and 4 PM) to enable them to plan their workload accordingly or raise any objections. Facilitators and presenters used a prompt card to structure the content of each round (Data S2). The named nurse of the patient chosen as the case study would present clinical details, history and current clinical state to a small group of critical care staff, facilitator and when possible, an observer to create an inquisitive dialogue between staff whilst formalising social and action learning in the workplace.

#### 2.2.2 Clinical Questions

A clinical question was defined as a matter requiring resolution or discussion that was specific to the clinical care of the patient (Tobiano et al. 2019). The group was encouraged to discuss the history and ask clinically relevant questions or challenge their assumptions. They then agreed to explore several of the clinical questions and highlight any possible knowledge gaps that could be addressed through the literature or local expertise (in situ). Approaches to answer the group's questions should be an iterative and flexible process depending on the types of questions generated.

#### 2.2.3 Choice of Patient Case Study

Two potential patient case studies were pre‐identified by the researcher or educator on the day of a round, based on learning opportunities offered, the education requirements of the group and expertise available. For example, this may relate to the type of inotropes used following cardiac arrest or lung protective ventilation strategies utilised in Adult Respiratory Distress Syndrome. The aim of the case study was to create an inquisitive dialogue between staff to identify areas of the patient case, which needed further examination through literature and evidence.

### 2.3 Participants

Participants volunteered to attend a round on an ad hoc basis. Each round generally consisted of:

- 3–4 nurses (this could be up to 7 staff depending on rota);
- one facilitator (educator, researcher, Lecturer Practitioner); and
- one observer for observational data collection.

Local members of the MDT (Education team, consultants, Advanced Clinical Practitioners‐ACPs), Physiotherapists) were invited to facilitate learning if questions generated by the group related to areas of their expertise.

### 2.4 Setting

The local NHS Trust constitutes three tertiary referral hospitals and one smaller hospital with 1500 beds and serves a population of over 600,000. The pilot study was conducted in a general, multilevel adult ICU consisting of two units across two sites, with a critical care bed capacity of 48. This unit has four side rooms per level, with most patients being cared for in a bay of 6–8 patients. Some patients would require 1:1 or 1:2 nursing care depending on acuity. Rounds were not to be conducted directly next to patients or relatives, but in a quieter section of the unit. Adaptations to the model, depending on the environment and the clinical pressures on that shift, were encouraged to maximise success and attendance.

### 2.5 Data Collection

#### 2.5.1 Phase 1 Observational Data Collection

Observational data were collected to provide an understanding of the impact of EBR, which may not be captured in survey responses alone. Round data were collected on a specifically designed electronic spreadsheet within Microsoft Excel [2022, version 16.66.1]. Observations followed two main processes. Firstly, the nominated observer would collect descriptive data, which were inputted into a bespoke electronic case report form. This descriptive data were prespecified with a view to being able to rigorously outline the structure of an EBR in service reports or publications. Prespecified data included:

- number of staff in attendance and their banding;
- date, start and finish times and duration of EBR;
- the number and types of questions generated;
- categories of impact (knowledge translation, patient care changes); and
- types of evidence accessed and required/use of local expertise (consultants, lecturer practitioners, ACPs, outreach librarians).

Secondly, the observer would make any other free text notes surrounding the utilisation and attendance of the EBR. Data were reviewed by the facilitator and observers to ensure accuracy. Whilst the free text notes were useful for the team to review and were a way to capture thoughts about the round more generally and contribute to overall process rigour, they did not form any part of the analysis described in this manuscript.

#### 2.5.2 Phase 2 Participant Survey

Each round was followed up with an email thanking staff for their participation and providing further resources such as presentations, papers, trials and websites to explore the clinical questions raised within the round. Staff were asked to complete a simple survey via a quick response code (Data S3) within Microsoft Forms [2024, Survey Microsoft Corporation]. This survey consisted of 14 questions that were based on the nursing round work by Tobiano et al. (2019). Five questions were Likert‐style questions, and the remainder were open‐ended questions to evaluate the round and learning. Some survey questions asked participants to highlight the impact of the round and select from a prespecified set, such as asking whether the round contributed to teamwork within the unit, confidence and ability to lead others, the identification of areas for practice improvement, and nurses' input into such improvements, ability to communicate clinical information, others' confidence and ability to lead. These impact questions were adapted from Tobiano et al. (2019) and staff were able to select multiple impact categories if they wished. Only minor changes were made to our survey with two additional questions added: ‘Did you feel supported?’ and ‘How might EBR be improved?’

Prior to use, the face validity of the survey was reviewed for understandability and appropriateness of each survey item by two members of staff including one senior lecturer practitioner, and one educator. The survey was peer reviewed by the nursing team, but no cognitive testing was conducted. Minor changes to the original survey were made such as grammar and wording. Some verbatim quotes will be used to support survey results presented.

### 2.6 Ethical Considerations

This service evaluation was reviewed by the clinical and senior nursing team and approved by the hospital NHS Clinical Governance department and allocated Ulysses Reference Number: 9077. Verbal consent was obtained from attendees to be observed and they were free to decline attending a round without consequence. No staff members refused to attend the round. Survey consent was implied by completing and returning responses. No personal patient data were collected as part of this service evaluation and patients were not directly observed.

### 2.7 Analysis

Survey responses to Likert‐type items such as the perceived efficacy of EBR, the selection of prespecified impact categories and demographic data were analysed using descriptive statistics in Microsoft Excel [2022, version 16.66.1] reporting percentages and frequencies. Observational data that included categorical (education content) and descriptive data (number of attendees, duration of the round) are presented with absolute and relative frequencies and percentages.

Data from the observations and the survey were analysed independently. Then, results were viewed together to give a multidimensional understanding of how well‐utilised the EBR were, the types of staff who attended, the rounds education content and how staff perceived the efficacy of this intervention. Open‐ended question responses were not qualitatively themed given the conciseness of staff responses but were mapped to support Likert scores and perceived impact categories.

## 3 Results

A total of 17 evidence‐based rounds were conducted with 83 clinical staff members between the pilot date range. Several staff attended; most were Band 5 nurses, whilst other roles included senior ICU staff, care support workers and nursing students. Full EBR descriptive statistics and attendee characteristics are described in Table 1.

**TABLE 1:** Evidence‐based round and attendee characteristics.

Descriptor	
Evidence‐based rounds (*n*)	17
Total number of attendees (*n*)	83
Mean (SD) number of attendees per round	5 (1.1)
Attendees banding *n* (%)	
Band 3	3 (3.6)
Band 4	0
Band 5	62 (74.7)
Band 6	10 (12)
Band 7	2 (2.4)
Student nurse	6 (7.2)

### 3.1 EBR Content and Delivery

Rounds lasted a mean of 39 min (SD 10.6 min) and a total of *n* = 77 clinically relevant questions were generated and explored with current evidence. Several key subject areas, dictated by the chosen patient case study, were explored during the EBR (Table 2).

**TABLE 2:** Evidence‐based Rounds questions and content.

Evidence‐based round structure	
Number of clinical questions generated (*n*)	77
Clinical subjects explored (*n*)	
Pancreatitis	3
Peritonitis	2
Intra‐abdominal hypertension	1
Sepsis	2
Renal replacement therapy	1
Therapeutic temperature management	2
Phaeochromocytoma	1
Aortic aneurysm repairs	1
Takasubo cardiomyopathy	1
Refractory asthma	1
Nitric oxide	1
Oesophagectomy surgery and complications	1

### 3.2 EBR Evaluation and Impact

**FIGURE 1:**
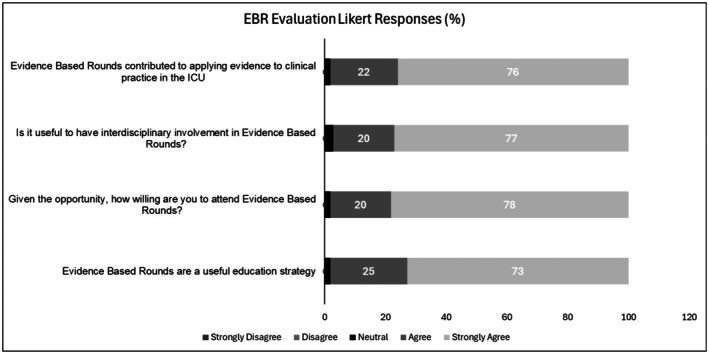
EBR Survey Likert responses.

Of the 83 staff who attended the EBR sessions, 55 completed an evaluation questionnaire resulting in a response rate of 66.3%. Most staff either agreed or strongly agreed that EBR was a useful education strategy (98%), that this model of ICU education contributed to applying evidence to clinical practice (98%) and would be willing to attend a future round (98%) (Figure 1). Free‐text responses supported these findings highlighting the perceived benefits of EBR, such as rounds being ‘Very helpful to link evidence‐based research to actual patients we are caring for’ *P8* and supporting the ‘Direct application of research with bedside care’ *P7*.

A small proportion of attendees (7/55, 12.7%) suggested that workload posed barriers to attending the EBR, which was captured through free text survey responses. Staff noted ‘Unfortunately I had to leave part way through due to an admission’ *P49*. However, several staff suggested that with adequate planning and adaptation to clinical pressures, this barrier could be mitigated: ‘Informed of time so I could plan care needs to ensure I was able to participate’ *P52*.

**FIGURE 2:**
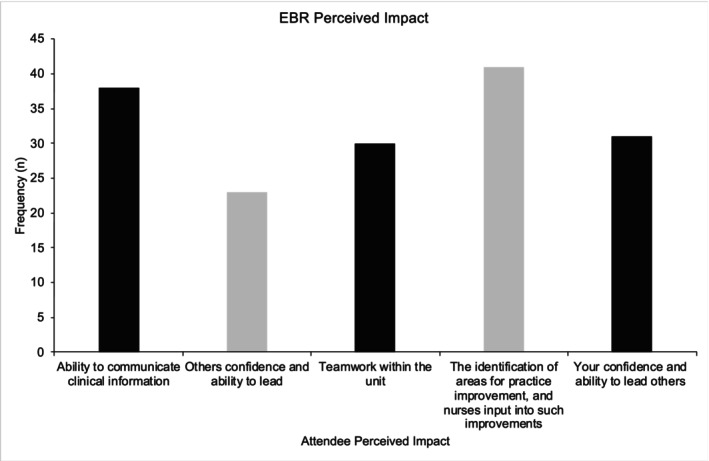
Commonly perceived personal or professional impact of EBR attendance.

Participants reported *n* = 163 categories of professional and personal impact arising from attending the EBR (Figure 2). The most common impact from EBR resulted in attendees who felt able to identify areas for clinical improvement (41 out of 163; 25.2%), enhanced their ability to communicate clinical information (38 out of 163; 23.3%) and increased their leadership self‐confidence (31 out of 163; 19.0%). Again, this was supported with the free text survey comments such as ‘It is a way to review how a patient was managed and identify other interventions that the patient may have from’ *P14,* ‘Providing evidence to support practice, asking questions and being able to discuss in a group ideas and experiences’ *P4* and ‘Participants are able to learn and ask questions that might lead to change in practice’ *P48*.

## 4 Discussion

In this study, we found that EBR was a positive learning experience for nurses and adaptable to a broad range of critical care curriculum and empirical clinical questions. This approach was effective in our local setting, enabling large numbers of attendees to receive education simultaneously, but with enough flexibility to address individual learner needs. We will discuss the pilot implementation of EBR by exploring workplace learning, its potential contribution to CPD, the model's perceived impact and its sustainability.

### 4.1 Workplace Learning

All staff who attended an EBR viewed it as a positive learning strategy and felt better able to communicate clinical information. Using real‐time patient cases and evidence‐based practice to guide workplace learning may better suit the learning styles of nurses, who are not passive recipients of information but instead value learning through practice (Tobiano et al. 2019). Such approaches are often referred to as active learning, which centres around participatory strategies. It is characterised by asking critical questions, being reflective and active engagement in self‐learning (Oliveira Silva et al. 2025). This is more aligned with clinical practice and the development of nursing expertise and may account for the additional benefits of evidence rounds seen in other studies such as higher workplace satisfaction (Aitken et al. 2011).

Although our evaluation did not directly measure clinical competence, it is important to recognise that learning through practice has been positively correlated with self‐reported competence among newly qualified nurses (Takase et al. 2015). This suggests that EBR may meaningfully and feasibly contribute more broadly to skill development in the critical care workforce and underpin patient safety improvements. The perceived impact data that was collected as part of this evaluation demonstrated that as well as learning, staff also felt that EBR contributed to their ability to communicate and felt more able to lead. Importantly, whilst outside the scope of this work, improvements in these domains have been shown in other studies to improve patient safety and reduce adverse events (Lee et al. 2023). Additionally, our study highlighted that staff perceived the EBR to support evidence‐informed practice and workplace learning, which may, in future studies, be explored in relation to patient‐level outcomes.

### 4.2 EBR as a Relevant CPD Model

Organisations should recognise that maintaining CPD opportunities within the workplace can help retain nurses who are building their careers, especially in times of financial restraints (Vázquez‐Calatayud et al. 2021). To be most effective, CPD should be aligned with individual requirements and that different forms of CPD learning should be considered at different stages of a nurse's career (Vázquez‐Calatayud et al. 2021). The EBR model addressed this by allowing staff to direct the content through collaborative questioning and discussions. Despite flexibility being advised within the literature, CPD is often conceptualised as formal education sessions, so more informal, diverse and creative education opportunities may not be explored by clinical education teams. This is particularly relevant when undergraduate nursing students are now exposed to and prefer a wide variety of educational methods, such as engaging and visual learning environments that integrate videos, stories, audio‐enhanced PowerPoint slides, simulations, group collaborative projects and discussion boards (Shorey et al. 2021). As these approaches become more common in the undergraduate curriculum, it is important to explore and utilise less traditional formats to maximise workplace learning once nurses are qualified.

The EBR model can support key workplace factors that facilitate professional development, including self‐motivation, positive culture, leadership, workplace learning and practice relevance, as identified in a rapid evidence synthesis of 39 studies (King et al. 2021). This is also highlighted in some of the feedback from staff where teachings from EBR provided a platform to improve teamworking and increase individual confidence to lead. The perceived impact data that were collected during this evaluation showed that the EBR education model specifically supported workplace learning and relevance to practice and may explain the positive uptake by staff observed during its pilot implementation in the critical care unit. This is also supported by a surprising finding from this evaluation, which was the voluntary EBR attendance by senior nursing staff despite the intended cohort being newer ICU nurses. Verbal feedback from senior nurses about the EBR model indicated that they felt invested in as well as enjoying this form of education. This emphasises the need for ongoing CPD support across all nursing grades and that the EBR model has the flexibility to address individual learner needs across all skill and experience levels.

### 4.3 Sustainability and Impact

The sustainability of education within critical care is an urgent service consideration. For interventions to be sustainable, they must be integrated into daily workflows, require minimal external resources, be perceived as valuable by staff and have buy‐in and support from unit managers and the wider organisation (Glasgow et al. 2019; Holtrop et al. 2021). Our findings suggest that the EBR model has several qualities that could provide sustainable ICU education and several broad benefits to the wider workforce. Firstly, EBR focusses on workplace learning, which aligns with national strategies to retain staff by supporting their professional development, particularly in the context of high turnover and educational gaps within ICU nursing (Critical Care Network Lead Nurses group 2022). Secondly, the EBR model encourages a pragmatic and flexible delivery based on staffing and workload, which is particularly relevant given the unpredictable nature of ICU environments. Finally, we were able to identify measurable EBR impact, the commonest being an improved ability to identify clinical areas for improvement. This mirrors the identified impact from the Tobiano paper, which implemented a similar initiative (Tobiano et al. 2019). Strongly evidencing these qualities in future evaluations would contribute to the sustainability of EBR and demonstrate undeniable value to hospital policy makers.

### 4.4 Limitations

There were some challenges to the delivery of EBR that should be considered. Not all EBR that were planned were successfully delivered due to unit acuity, educator time availability, or a suitable patient. Also, some suitable patients were in single‐side rooms, which meant having many staff at the bedside was logistically difficult and not appropriate. We also acknowledge that the clinical setting and the small number of sessions held may not be generalisable to other clinical areas due to workflows and workloads. This service evaluation was conducted on a single site and so the implementation of EBR should be considered at a local level and actively adapted to the local context and workflows to ensure its success. This is also a service evaluation and therefore not deemed research, so there are opportunities for future multi‐site research studies examining the implementation of EBR on a larger scale.

The number of rounds and participants reported in this paper is small and so the data in this work were examined for descriptive purposes only, but it may be possible to conduct larger studies with adequate power to examine statistical significance. We did not capture any patient‐focussed outcomes, which could be considered a limitation as this study mainly focusses on staff perceptions and perceived learning outcomes from EBR. In future longitudinal studies, consideration of patient satisfaction and patient‐centred outcomes should map to the organisational trust‐wide agenda as well as the 10‐year Health Plan for England to demonstrate the impact of EBR (NHS England 2025). There was no qualitative theming of open‐ended responses as these were pre‐specified as described in the work reported by Tobiano and Dale (2022). However further in‐depth qualitative analysis may have been of benefit. Finally, and importantly, EBR were delivered by the existing education team as this was felt to be most appropriate given their skill sets and understanding of the local workforce education needs. However, it is essential to explore and acknowledge the potential influence on responses this may have had despite surveys being anonymous and via an electronic system. Staff may have felt beholden to give positive feedback, which may over inflate positive results and not be a true representation of the overall impact of EBR.

## 5 Conclusions

In our setting, the EBR model was perceived to be a feasible and effective workplace education approach. Embedding EBR into regular practice, rather than treating it as an isolated educational event, may promote a culture of continuous learning and is a hypothesis that warrants further testing. Our work showed that staff perceived the EBR to support evidence‐informed practice and workplace learning, which may, in future studies, be explored in relation to patient‐level outcomes. Whilst EBR were feasible during this pilot, long‐term success will depend on management buy‐in, allocation of protected time, and integration into existing education frameworks.

## Funding

The authors have nothing to report.

## Ethics Statement

This QI project was registered and approved within the Trust's governance system and allocated Ulysses Reference Number: 9077. All staff were notified that survey responses may be used for publication and were given the option to have their data removed from the analysis. No participants withdrew their data.

## Consent

The authors have nothing to report.

## Conflicts of Interest

The authors declare no conflicts of interest.

## Supporting information


**Data S1:** SQUIRE Reporting checklist.

**Data S2:** EBR.

**Data S3:** EBR evaluation survey.

## Data Availability

Data is available upon request from the authors.
