# Supplementary material for: A Descriptive Evaluation of Evidence‐Based Rounds in Critical Care Using Mixed Data Types
Source: J Adv Nurs. 2025 Dec 12;82(8):8116–23. doi: 10.1111/jan.70420 (PMC13356355; doi:10.1111/jan.70420)
Supplement: Supplementary file 2 — Data S2: EBR. [file JAN-82-8116-s001.docx]

EBR Supplementary File 2

Evidence-Based Rounds Facilitator Prompt Card

**Aim**

To facilitate an inquisitive discussion between OCC nursing staff members through case-based learning which generates clinically important questions and creates an opportunity to:

- consolidate clinical understanding
- refine patient assessment skills
- refine presentation skills
- identify knowledge gaps
- learn from current evidence
- advance critical thinking and problem solving
- underpin clinical understanding for competency completion
- provide a safe learning experience supporting staff development
- enhance patient care delivery

**Staff**

1 Facilitator

1 Observer

3-4 OCC staff members

**Evidence-Based Round Structure**

1. Named nurse presents patient using a systematic approach (10 mins)
   1. Name and age
   2. Presenting complaint
   3. Past medical history
   4. Community medication history
   5. Reason for ICU admission (organ support, CVVH, ventilation)
   6. Key significant events since admission
   7. Summary of A-E assessment and nursing diagnosis/identification of current progress and issues
2. Open to group to formulate questions and clarify points (all is captured by observer) (5 mins). Types of question may be:
   1. What is the evidence base for this treatment?
   2. What are the key concerns for this patient?
   3. What is our current compliance with the care bundles for this patient? VAP, Tidal Volumes, feeding, rehab, sedation holding, lines care, weaning, delirium care, sleep, fluid status,
   4. What is working and what is not?
   5. Why are we doing x rather than y?
3. Group agrees on a final question which is clinically significant
4. Facilitator/observer addresses that question using evidence, resources, local expertise (Education team, consultants, ACPs, dieticians, physios, research team) (20 mins)
5. The group agree on outcomes, actions, impact of this EBR (call to action or **One Key Act**).
