# Supplementary material for: A Descriptive Evaluation of Evidence‐Based Rounds in Critical Care Using Mixed Data Types
Source: J Adv Nurs. 2025 Dec 12;82(8):8116–23. doi: 10.1111/jan.70420 (PMC13356355; doi:10.1111/jan.70420)
Supplement: Supplementary file 3 — Data S3: EBR evaluation survey. [file JAN-82-8116-s002.docx]

Supplementary File 3 EBR Evaluation Survey

**Evidence Based Rounds Survey**

What is your age? (not obligatory)

1. What is your highest qualification?

2. Did you present a patient in the Evidence Based Round or facilitate?

3. What helped and hindered your ability to participate in Evidence Based Rounds?

4. What are the perceived benefits, if any, of Evidence Based Rounds?

5. Did you feel supported in the process? (Yes/No/Maybe)

6. Was it a positive learning experience? (Yes/No/Maybe)

7. Did it change the way in which you might present clinical information to medical and allied health team members? (Strongly Disagree to Strongly Agree scale)

8. Evidence Based Rounds are a useful education strategy (Strongly Disagree to Strongly Agree scale).

9. Given the opportunity, how willing are you to attend Evidence Based Rounds? (Strongly Disagree to Strongly Agree scale)

10. Is it useful to have interdisciplinary involvement in Evidence Based Rounds? (Strongly Disagree to Strongly Agree scale)

11. Evidence Based Rounds contributed to applying evidence to clinical practice in the ICU (Strongly Disagree to Strongly Agree scale).

12. Can we improve the Evidence Based Rounds model in any way?

13. Has participation in Evidence Based Rounds influenced (tick all that apply):

- Teamwork within the unit

- Your confidence and ability to lead others

- The identification of areas for practice improvement, and nurses’ input into such improvements

- Ability to communicate clinical information

- Others' confidence and ability to lead
